# Longitudinal assessment of cognitive function in testicular cancer patients prior to orchiectomy and 9 months later and associations with tumor markers

**DOI:** 10.1007/s00520-025-09637-w

**Published:** 2025-06-17

**Authors:** Niels Fog Højris, Yoon Frederiksen, Mads Agerbæk, Solvej Heeringa Nielsen, Mick Holt, Signe Lehn Brand, Nikoline Lysemose Petersen, Ulla Breth Knudsen, Ali Amidi

**Affiliations:** 1 Fertility Clinic, University Clinic Horsens, Horsens, Denmark; 2 The Sexology Unit, Aarhus University Hospital Psychiatry, Aarhus, Denmark; 3 Department of Clinical Medicine, Aarhus University, Aarhus, Denmark; 4 Department of Oncology, Aarhus University Hospital, Aarhus, Denmark; 5 Department of Psychology & Behavioural Sciences, Aarhus University, Aarhus, Denmark; 6 Unit for Psychooncology & Health Psychology, Department of Psychology & Behavioural Sciences, Aarhus University, Aarhus, Denmark

**Keywords:** Testicular cancer, Cognitive function, Memory, Orchiectomy, Cancer-related cognitive impairment

## Abstract

**Purpose:**

Research shows that testicular cancer patients (TCPs) evidence cognitive impairment (CI) in the absence of systemic therapy, suggesting that the cancer itself or surgery may play a role. In the present study, we undertook longitudinal cognitive assessments in TCPs from pre- to post-orchiectomy.

**Methods:**

Enrolled TCPs underwent cognitive assessment with the Cambridge Neuropsychological Test Automated Battery prior to orchiectomy (T1) and 9 months later (T2). Test outcomes were norm-adjusted and converted to *z* scores. A mean global composite score (GCS) across all tests was calculated. A standardized regression-based approach was used for the longitudinal analyses. Biological markers, including lactate dehydrogenase (LDH) and alpha-fetoprotein (AFP), were also assessed.

**Results:**

Of 48 eligible patients, 29 (60%) participated and 20 (69%) underwent follow-up assessment. Mean *z* scores (SD) at pre-orchiectomy ranged from − 0.16 (0.73) to 0.53 (0.76). GCS was 0.26 (0.64) with three TCPs (10.3%) evidencing clinically significant CI. Mean standardized change *z* scores from pre- to post-orchiectomy ranged from − 0.42 (0.87) to 0.67 (0.76). A statistically significant decrease in GCS was observed from T1 to T2 (*p* = .03). Statistically significant associations were observed between LDH and several cognitive domains (*r*’s = − .48 to − .52), and between AFP and executive function (*r* = − .44).

**Conclusion:**

Overall, the prevalence of CI was low, and cognitive performance from pre- to post-orchiectomy was within a normative range. However, a decrease in overall cognitive function was noted, and a possible association was observed between cognitive performance and LDH and AFP.

Trial registration.

ClinicalTrials.gov Identifier: NCT03880994.

## Introduction

Testicular cancer (TC) is the most common malignant tumor among young men in developed countries [1]. TC is preceded by a focal maturation defect in germ-cell precursors during embryogenesis. Although the etiology is still unknown, some associated or predisposing factors have been discovered including family history, cryptorchism, reduced semen quality, and previous TC in the contralateral testicle [2].

The primary treatment for localized TC (stage I) is orchiectomy followed by surveillance. Depending on stage, chemotherapy or radiotherapy following surgery is also an option [2]. Standard first-line chemotherapy is a combination of cisplatin, bleomycin, and etoposide (BEP) and is known to have peripheral neurotoxic side effects [3]. Overall, TC has a very low mortality rate, and after introduction of cisplatin-based chemotherapy, the cancer specific 5-year survival is above 97% [4, 5], and patients have a long life expectancy after TC treatment. This makes immediate and long-term side effects and sequelae particularly important in this patient group.

Cancer-related cognitive impairment (CI) is an important aspect to consider when treating TC patients (TCPs) and cancer patients in general [6]. Previously, chemotherapy was believed to be the primary cause of CI in cancer patients. However, it is now widely debated whether CI is a consequence of the cancer itself, surgery, anesthesia, antihormonal or radiation therapies, psychological and behavioral factors, or a multifactorial combination of these factors [6–13]. A common characteristic of these factors is that they may induce neuroinflammation that can result in CI [14]. Studies suggest an association between the proinflammatory cytokine TNF-Alpha and CI in breast cancer populations [15, 16], and such an association has also been observed in TC patients [8]. Chemotherapy-related neurotoxicity indirectly caused by proinflammatory cytokines has also been suggested as a pathophysiological mechanism with the cytokines affecting dopaminergic and cholinergic pathways [17]. Plasma cytokines may reach brain cells either by crossing the blood–brain-barrier directly in circumventricular regions [18], through active transport [19], or by increasing the brain cells’ own synthesis of cytokines [18].

The number of studies investigating CI in TCPs has been increasing, and most studies have shown that CI is common among TCPs. However, it remains unclear whether CI is associated with chemotherapy, surgery, or the cancer disease itself [8, 10, 20–23]. A growing body of evidence has shown that CI is present already prior to chemotherapy [9, 11], which is consistent with other studies that have not been able to find differences in cognitive performance between surgery only and chemotherapy-treated patients [20, 22, 24]. One study found a tendency toward a greater decline in the chemotherapy group compared to the surgery-only group [8], and another reported greater rates of cognitive decline at a 12-month follow-up, comparing chemotherapy-treated patients with those who underwent surgery-only, in a dose-dependent manner [23]. Recently, a study of long-term survivors of TC showed a significant impaired performance in TCPs irrespective of treatment compared with healthy matched controls [25]. The research field is further complicated by the fact that orchiectomy is usually initiated very shortly after the detection of the cancer, and thus, reports of cognitive function prior to surgery is currently lacking.

The tumor markers alpha-fetoprotein (AFP), human chorionic gonadotropin (HCG), and lactate dehydrogenase (LDH) are widely recognized as tumor markers, and their levels have been correlated with cancer prognosis [26]. Serum levels of LDH is an unspecific marker of cellular proliferation and decay, and as such also used as an unspecific serum marker of testicular cancer. LDH is elevated in 30–60% of TC cases and associated with histology and tumor burden [26, 27]. Associations between LDH levels and cognitive impairment have earlier been reported in other populations [28–30], and it has been suggested that LDH may be a marker of inflammation that could lead to CI [29]. Associations between LDH and CI have not previously been investigated in TC populations.

Beyond CI, there are other concerns regarding psychosocial late effects with reports of increased prevalence of anxiety among long-term survivors of TC [31, 32]. However, the reported prevalence in earlier studies is often measured years to decades after cancer treatment [31], and, thus, it is unknown if these reports represent pre-existing symptoms that are perpetuated.

In the present study, we aimed to investigate cognitive function in newly diagnosed TCPs prior to orchiectomy and 9 months after. To the best of our knowledge, cognitive function has not previously been investigated in a TC population prior to surgery. Furthermore, we aimed to explore possible tumor-related correlates of cognitive function.

## Methods

### Participants

Men newly diagnosed with TC who were referred to the fertility clinic in Horsens, Denmark, for semen cryopreservation prior to orchiectomy were recruited from March 25, 2019, to June 2, 2021.

The inclusion criteria were men aged 18–45 years with a recent diagnosis of TC referred to cryopreservation of semen prior to treatment at the fertility clinic in Horsens, Denmark. Patients who were not able to speak, read, or write Danish or who were not able to give informed consent were excluded.

### Procedure

Recruitment was conducted by medical doctors at the fertility clinic during the patients’ first visit. A neuropsychological assessment and a questionnaire package regarding demographic and psychological variables were undertaken at baseline in the fertility clinic prior to orchiectomy (T1). The same tests and questionnaire package were administered at a 9-month follow-up assessment (T2) in the fertility clinic, Aarhus University Hospital or at facilities at the Department of Psychology, Aarhus University.

### Neuropsychological assessment

Cognitive performance was assessed with the validated Cambridge Neuropsychological Test Automated Battery (CANTAB) Connect Research software [33]. The assessment was performed using a tablet validated for the purpose. Patients completed six different cognitive tests to the best of their abilities. Each test took from 2 to 15 min, and the total test time was approximately 45 min. Precautions were made to make the test circumstances as standardized as possible. Testing took place in a quiet room, and the tests were administered by trained healthcare personnel under the supervision of a senior expert in neuropsychology (AA). The patients were not served coffee or other neuro-stimulating substances prior to testing. Testing took place at the fertility clinic, where patients had been referred for semen storage. Multiple cognitive domains were assessed: *Reaction time* was assessed with the reaction time test (RTI); *sustained attention* with the rapid visual information processing test (RVP); *working memory and strategy* with the spatial working memory test (SWM); *executive function* with the one touch stocking of Cambridge test (OTS); *visual episodic memory* with the paired associates learning test (PAL); and *visual working memory* with the spatial span test (SPS)*.*

### Self-reported outcomes

The questionnaire package consisted of validated scales. In the present study, the following were included: The Hospital Anxiety and Depression Scale (HADS) to assess symptoms of depression and anxiety, and the WHO-5 Well-being Index to assess quality of life. Demographic questions were also part of the questionary package.

### Biological variables

Biochemical measurements from blood samples drawn as part of standard clinical practice were attained through patient’s medical records. The serum tumor markers alpha-fetoprotein (AFP, µg/L), human chorionic gonadotropin (HCG, IU/L), and lactate dehydrogenase (LDH, U/L) were measured within 2 weeks prior to planned surgery. Hormonal serum markers included follicular-stimulating hormone (FSH, IU/L), lutein hormone (LH, IU/L), testosterone (T, nmol/L), and sexual hormone-binding globulin (SHBG, nmol/L) and were measured 2–10 months after surgery.

### Statistical analysis

Scores from the respective cognitive tests at baseline, except for RKI, were compared to normative data from the CANTAB software with adjustment for age, gender, and education level [33]. Each principal test outcome was calculated as a *z* score denoting the patient’s performance within the normal distribution in standard deviations from the mean. For each patient, a *z* score ≤ − 1.5 on a single test was considered indicative of significant CI within the respective cognitive domain. However, to prevent false positive categorization, overall CI was only indicated if a patient had a *z* score of ≤ − 2.0 on one test, or ≤ − 1.5 on two or more tests, in line with recommended guidelines [15, 28]. Across the five tests where comparison to normative data were possible, an estimate of overall performance was calculated as a mean *z* score termed global composite score (GCS). For the prospective analysis of cognitive performance data, a standardized regression-based (SRB) approach accounting for retest effects and age was used to assess change in cognitive performance across time using published regression-based change equations for the CANTAB [34]. Such equations were available for all test outcomes expect for the OTS. The SRB approach results in a change *z* score with negative values indicating lower cognitive performance than normatively expected when considering the retest effect. At T2, a global composite change *z* score was calculated as the average of the available change *z* scores, which included all tests except for the OTS. For the HADS, a score of ≤ 8 was categorized as exhibiting anxiety or depression in line with previous studies [35]. Within-group changes on cognitive outcomes and psychological variables from T1 to T2 were tested with bootstrapped paired-sample *t* tests. Associations between standardized cognitive test scores and biomarkers and symptoms of anxiety and depression were explored with boot-strapped Pearson’s correlation tests. All statistical analyses were performed using SPSS 28.0 [36] with the statistical significance level set to *p* < 0.05.

## Results

### Participants

Of a total of 48 eligible patients, 30 (62.5%) accepted enrollment. One patient was later excluded due to histology showing that the testicular tumor to be non-malignant, leaving 29 for analysis at T1. Twenty patients completed the 9-month follow-up assessment, resulting in a dropout rate of 31.0%. Due to technical issues, one subject did not complete the questionnaires, and therefore, demographic and psychological measures are lacking for this subject. Mean age of all enrolled patients was 30.5 (SD = 7.07). The majority of patients were diagnosed with a seminoma (*n* = 17, 58.6%). Mean time from test to orchiectomy was 7.9 days (SD = 8.9), with a median of 2 days. Mean time from T1 to T2 was 289.6 days (SD = 38.5), with a median of 283 days. A total of 11 patients had stage 2 or 3 disease. Among these seven patients received three series of bleomycin, etoposide, and platinum (BEP), one patient received 2.5 series of BEP, one patient received two series of paclitaxel, ifosfamide, and cisplatin, and one patient received a single series of BEP combined with radiation therapy. Finally, one patient was treated with radiotherapy only. Of the participants who completed follow-up assessments, only five patients had received three series of BEP. For other demographic and clinical characteristics please, refer to Table 1.

**Table 1:** Demographic, clinical, and psychological characteristics of the sample

**Demographic characteristics**	Mean (SD)/*N* (%)	Median
Age	30.5 (7.07)	29
Education, highest completed education *N* (%) Primary school Secondary school Graduate degree	2 (7) 14 (50) 12 (43)	
Annual income (in 100.000 DKK) (mean, median, SD)	3.75 (2.80)	3
Marital status, *N* (%) Single/not cohabiting Married/cohabiting	11 (39) 17 (61)	
Exercise (hours/week)	6.2 (6.68)	5
Body mass index	24.99 (3.38)	24.5
Alcohol consumption (drinks/week)	5.3 (5.90)	3
Smoking status Yes No	3 (11) 25 (89)	
**Clinical characteristics**	Mean (SD)	Median
Histology, *N* (%) Seminoma Non-seminoma	17 (59) 12 (41)	
Stage, *N* (%) S1 S2 S3	18 (0.62) 7 (0.24) 4 (0.14)	
Orchiectomy at T2, *N* (%) Unilateral	29 (100)	
Chemotherapy at T2, *N* (%) Bleomycin, etoposide, platinum Paclitaxel, ifosfamide, cisplatin	9 (31) 1 (3)	
Radiotherapy at T2, *N* (%)	2 (7)	
Tumor markers AFP, µg/L HCG, IU/L LDH, U/L	48.2 (197.7) 1000.9 (4508.3) 245.3 (148.7)	3.0 2.0 200
Hormone levels (post-surgery) FSH, IU/L LH, IU/L Testosterone, nmol/L SHBG, nmol/L	12.19 (9.76) 8.11 (3.03) 14.92 (5.19) 33.56 (12.47)	7.6 7.8 14.2 31.5
**Psychological characteristics**	Mean (SD)	*p* value
HADS – anxiety Time 1 Time 2	7.32 (4.40) 4.63 (2.97)	.018
HADS – depression Time 1 Time 2	2.95 (2.88) 3.37 (4.28)	.64

*AFP*, alpha-fetoprotein; *HCG*, human chorionic gonadotropin; *LDH*, lactate dehydrogenase; *FSH*, follicular-stimulating hormone; *LH*, lutein hormone; *SHBG*, sexual hormone-binding globulin; *HADS*, hospital anxiety and depression scale; *SD*, standard deviation

### Neuropsychological performance

At baseline, 10.3% (*n* = 3) evidenced CI, with mean *z* scores ranging from − 0.16 to 0.44. At T2, 5.0% (*n* = 1) evidenced cognitive impairment (please refer to Table 2), while the SBR change score ranged from − 0.42 to 0.54. Within-group analysis revealed no statistically significant changes in cognitive performance from T1 to T2 in any specific cognitive domain. However, a statistically significant decrease was observed for the global composite score from T1 to T2 (*p* = 0.03). No between-group differences in neuropsychological test performance were observed between patients who had received chemotherapy at T2 versus those who had not (all *p*’s > 0.11).

**Table 2:** Neuropsychological test-scores at baseline (T1) and follow-up (T2)

		*N* = 29		*N* = 20		*N* = 20^c^
Cognitive domain	CANTAB test	T1 –*z* score mean (SD)	T1 – impairment frequency (*z* ≤ − 1.5) *N* (%)	T2 change *z* score mean (SD)	T2 – impairment frequency (*z* ≤ − 1.5) *N* (%)	*p* value T1-T2
Reaction time	RTI (ms)	356.7 (35.38)	-	− 0.42 (0.87)^a^	1 (5.0)	-
Sustained attention	RVP	− 0.16 (0.73)	1 (3.4)	0.07 (1.08)^a^	1 (5.0)	.84
Working memory and strategy	SWM	0.31 (1.42)	3 (10.3)	0.46 (0.48)^a^	0 (0)	.70
Executive function	OTS	0.53 (0.76)	1 (3.4)	0.67 (0.76)^b^	1 (5.0)	.80
Visual episodic memory	PAL	0.170 (1.15)	3 (10.3)	− 0.22 (0.52)^a^	1 (5.0)	.07
Visual working memory	SPP	0.44 (1.20)	1 (3.4)	0.19 (0.78)^a^	1 (5.0)	.59
GCS		0.26 (0.64)	-	0.02 (0.33)^a^	^−^	.03*
Overall CI *N* (%)			3 (10.3)	-	0 (0)	-

*RTI*, reaction time test; *RVP*, rapid visual information processing test; *SWM*, spatial working memory test; *OTS*, one touch stocking of Cambridge test; *PAL*, paired associates learning test; *SPS*, spatial span test; *GCS*, global composite score; *SD*, standard deviation; *CI*, cognitive impairment. ^a^Change *z* score based on the standardized regression-based approach. ^b^*z* score based on CANTAB normative data. ^c^Statistical analysis performed for participants with available data from both time points. *Statistically significant at *p* < .05

### Psychological distress and associations with cognitive function

HADS anxiety score was 7.32 (SD = 4.40) at T1 and 4.63 (SD = 2.97) at T2 indicating an overall decrease in anxiety levels across time (*p* = 0.018). The prevalence of substantial anxiety at T1 was 50.0% (*n* = 14) and 15.0% (*n* = 3) at T2. HADS depression score was 2.95 (SD = 2.88) at T1 and 3.37 (SD = 4.28) at T2 with no significant changes observed across time. The prevalence of high levels of depression symptoms at T1 and T2 was 14.3% (*n* = 4) and 15.0% (*n* = 3), respectively. Depression at baseline was significantly correlated with slower reaction time (*p* = 0.013) and poorer OTS score (*p* = 0.005). No associations were observed with cognitive performance at follow-up. Depression and anxiety symptoms at baseline were significantly correlated with lower scores on the WHO 5 wellbeing index (*p*’s < 0.001).

### Tumor markers and associations with cognitive function

**Fig. 1:**
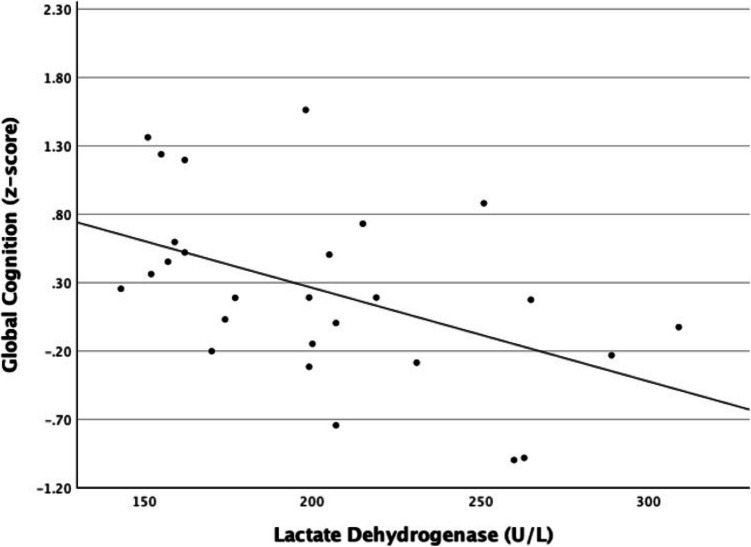
Association between global cognition and lactate dehydrogenase

The average level of LDH was 237U/L (SD = 148.7) and the median 200 U/L, though there were two outliers with values of 956 and 434. Correlation analysis showed a moderate association between LDH concentrations and baseline cognitive performance with higher levels of LDH being associated with lower scores on OTS test (see Table 3). However, when excluding the two extreme LDH outliers, the results were statistically significant for RVP, PAL, and GCS (see Fig. 1). The average level of AFP was 48.2 µg/L (SD = 197.7) and the median 3. Three outliers with the values 1070, 58, and 82 µg/L were observed. When excluding these outliers, correlation analysis showed a moderate significant association between AFP concentrations and baseline cognitive performance with higher levels of AFP being associated with lower scores on OTS test. No associations were found between cognitive function and serum HCG. No associations were observed between cognitive function and gonadal hormones FSH, LH, and T, or SGBH (data not shown). Likewise, no association were observed between LDH and symptoms of anxiety or depression.

**Table 3:** Associations between pre-surgery (T1) tumor markers and cognitive function

*N* = 22	**RTI**	**RVP**	**SWM**	**OTS**	**PAL**	**SSP**	**GCS**
LDH (U/L)	.177	−.517**	−.069	−.520**	−.487**	−.095	−.475*
AFP (µg/L)	.185	−.357	.215	−.435*	−.382	−.161	.170
HCG (IU/L)	−.036	.013	.214	.036	.339	.200	.314

*LDH*, lactate dehydrogenase; A*FP*, alpha-fetoprotein; *HCG*, human chorionic gonadotropin. *Statistically significant at *p* < .05. **Statistically significant at *p* < .01

## Discussion

To the best of our knowledge, the present study is the first to assess cognitive function in newly diagnosed TCPs *prior* to orchiectomy, with a subsequent follow-up assessment 9 months *after* orchiectomy. Overall, our results revealed that TCPs did not evidence a high level of CI prior to orchiectomy. An impairment prevalence of 10% is relatively low compared with other published studies showing rates ranging from 34 to 65% [9, 10, 23, 37]. For example, we have previously shown a high prevalence of cognitive impairment (58%) in recently diagnosed TCPs prior to chemotherapy [9]. Wefel et al. [37] found a similar high prevalence (46%) in their sample of TCPs. However, it should be noted that these studies mainly investigated CI *after* orchiectomy, which could point to either the cancer disease itself or the orchiectomy as risk factors for CI. Furthermore, these studies used more traditional pen and paper tests and compared TCPs with healthy controls. Our study adds to the literature by showing that pre-orchiectomy CI is low, which may suggest that the cancer disease itself is less likely to be the cause of cognitive alterations at this stage. Instead, it has been hypothesized that surgery and associated factors may explain CI at this early stage of the cancer trajectory. For example, surgery and anesthesia have been shown to be related to postoperative CI among young surgery patients aged 18–39 years [38]. Surgery alone may be a cause of CI due to surgery-induced inflammation that could activate central pro-inflammatory cytokines leading to neural damage [12, 39]. Furthermore, even minor surgical procedures have been associated with CI [40]. Previously reported CI after surgery in TCPs could, thus, partly be due to the surgery and/or anesthesia. However, the prospective results of the present study did not initially confirm this as changes on most cognitive tests from pre- to post-orchiectomy remained within normative ranges and were not statistically significant. One exception, however, was the global cognition score. There could be several reasons for this. First, in the present study, we used digital and tablet-based tests to assess cognition, whereas previous studies have mainly used pencil-and-paper assessment methods. Paper and pencil test could be more sensitive to capture cognitive changes in this population, although computerized test has shown to be equally sensitive in populations with mild cognitive impairment [41]. Second, the inclusion rate of 63% may have introduced a risk of selection bias as patients experiencing CI prior to surgery could have potentially been less prone to participate. Finally, a dropout rate of 31% from baseline to post-orchiectomy could also introduce additional bias, although the level of drop-out is relatively comparable to other studies of cancer-related cognitive impairment. One reason for this could be that the second assessment was undertaken at another site than the first assessment, which could have posed logistical challenges for some participants in terms of completing both assessments. To explore whether patients who dropped out were systematically different in term of their cognitive performance, we compared baseline test scores of those who dropped out, with those who did not. We found that out of the seven cognitive outcomes, only one test score related to executive function was significantly lower in the dropped out patients but still remained within an expected range.

As mentioned, we did observe a mean reduction in global cognition from T1 to T2. While this result is in accordance with previous studies showing a decline in global cognition across time in TCPs receiving cisplatin-based chemotherapy [8], other studies have not observed such a decline in global cognition but instead in specific cognitive functions [10]. In the present sample, five patients had received BEP chemotherapy at the T2 follow-up assessment. However, we observed no significant differences between these patients and those who had not received chemotherapy. It is important to note that the GCS did decline even though the frequency of participants with overall CI was very low at T2.

Multiple factors could influence patients’ cognition, such as fatigue, distress, medication, and socioeconomic status [42, 43]. In the present study, cognitive testing was performed few days after TC diagnosis, and therefore, diagnosis-related distress could possibly have influenced patients’ cognitive function [44]. While previous studies have reported mixed results regarding the association between CI and symptoms of anxiety and depression [9, 11, 20], we did observe associations between higher baseline depression and worse reaction time and executive function Psychological distress in the current sample of TC patients was generally high. Approximately 50% of participants reported high levels of anxiety prior to surgery, which is expected given the early stage of the cancer treatment trajectory. At follow-up, this rate had reduced to 15%, which is in line with previous studies reporting rates of 20% [31, 32].

Strength of the present study includes adjustment for retest effects of the neuropsychological testing, as well as the inclusion of tumor biomarkers. We found significant associations between cognitive performance and LDH and AFP, which has not previously been reported among TCPs. A link between LDH and cognitive performance has, however, been shown among children with sickle cell disease and patients undergoing cardiac surgery [28–30]. Such an association may be mediated by inflammation, most notably pro-inflammatory cytokines, which have been hypothesized to induce neuronal damaging in breast cancer populations [15, 16]. For AFP, a plausible explanation could be that AFP-levels are associated with tumor burden and, thereby indirectly CI as with LDH.

The present study also has limitations that need to be considered. First, given the low number of new cases of TC in Denmark per year, together with the fact that not all new TC patients require semen depositing at a fertility clinic, the current sample size was relatively small limiting statistical power and generalizability of the results. Second, we did not include a healthy control group which would have allowed us to more accurately model the retest and practice effects in the specific context.

From a patient care perspective, our results highlight the importance of pre-surgical counseling to set expectations regarding potential cognitive changes. Given the observed decline in global cognition, clinicians could consider incorporating cognitive assessments into the routine follow-up of TC patients. Additionally, our findings suggest that psychological factors, such as anxiety, are prevalent in TC patients pre-surgery but tend to decrease over time. This underscores the need for psychosocial support interventions, especially in the early phases of diagnosis and treatment.

For future research, our study raises several important questions that warrant further investigation. First, while we found associations between cognitive performance and tumor markers (LDH and AFP), the underlying mechanisms remain unclear. Future studies should explore the role of inflammation, neurotoxicity, and hormonal changes in cognitive function among TC patients. Second, a larger, multi-center study with a matched healthy control group would help clarify the long-term trajectory of cognitive changes and differentiate cancer-related cognitive impairment from other contributing factors such as stress and surgery. Finally, future research could explore interventions aimed at mitigating cognitive decline in TC patients, such as cognitive training, lifestyle modifications, and pharmacological approaches targeting neuroinflammation.

Taken together, our results suggest that the frequency of CI in TCPs prior to orchiectomy is low, and the level of cognitive function is within a normative range. This may indicate that surgery or anesthesia is primarily responsible for the high frequency of CI that has previously been shown among TCPs after orchiectomy. However, in the present study, we did not observe substantial CI levels in TCPs at nine months after orchiectomy, which could potentially be due to methodological differences. We showed that anxiety is highly present in young TCPs prior to treatment but decreases significantly after nine months. Associations between cognitive function and LDH and AFP have only been sparsely described in previous literature and await further replication in future research.

## Data Availability

All data generated and analyzed during this study are with the principal investigator of the study. Data can be provided upon reasaniable requests.
